# Acquired uterine arteriovenous malformation following dilation and curettage: a case report

**DOI:** 10.11604/pamj.2022.42.71.35371

**Published:** 2022-05-26

**Authors:** Andela Manisha, Amardeep Tembhare

**Affiliations:** 1 Department of Obstetrics and Gynecology, Jawaharlal Nehru Medical College, Datta Meghe Institute of Medical Sciences (Deemed to be University), Sawangi (Meghe), Wardha, Maharashtra, India

**Keywords:** Uterine arteriovenous malformation, uterine artery embolization, dilatation and curettage, power Doppler, case report

## Abstract

Arteriovenous malformation of the uterus is a rare encounter with sporadic incidence. They are usually acquired following events like dilation and curettage or abortions. It should always be considered as a diagnostic possibility in women presenting with torrential vaginal bleeding. We report a case of 24-years-old woman presenting with excessive bleeding following dilation and curettage. She was diagnosed with an acquired uterine arteriovenous malformation after conducting Doppler angiography. She was meticulously managed by performing uterine artery embolization. Embolization technique for uterine arteriovenous malformation (AVM) is generally used in fewer crisis circumstances as well as in emergency situations. Management by selective arterial embolization reduces the morbidity of surgery and hospital stay.

## Introduction

Uterine arteriovenous vascular malformation (UAVM) results from abnormal communication between the arteries and veins of the uterus. It is an uncommon condition, which becomes potentially life endangering situation due to excessive bleeding. Uterine arteriovenous vascular malformation is further divided into congenital or acquired [1]. Congenital AVMs have been found to have a central nidus which contain blood vessels consisting of both arteries and veins. It consists of various feeding arteries and draining veins. These various kinds of vessels which are involved may differ in their calibre. Acquired AVMs are found to be associated with inflammation, infection, retained products of conception (RPOC), gestational trophoblastic disease (GTD), pelvic trauma, gynecological malignancies, and exposure to dimethyl stilbestrol [2,3]. Acquired malformations usually follow events like pregnancy and are diagnosed with conditions like profuse uterine bleeding despite following medical measures. These malformations resulted in bleeding which is episodic and could become torrential, requiring hospital admission followed by multiple blood transfusions. Previously, the mainstay of treatment was considered to be hysterectomy, but with the technological advancement during the modern era, conservative management is now made available [4]. Numerous non-invasive diagnostic techniques have been suggested to diagnose uterine AVM, such as contrast-enhanced computed tomography (CECT), magnetic resonance imaging (MRI), and color flow Doppler ultrasound [5]. With the advancements in three-dimensional (3D) ultrasound, power Doppler angiography, or amplitude-mode color Doppler ultrasound, may provide another non-invasive and more specific diagnostic approach.

## Patient and observation

**Patient information:** a 24-year-old female presented to the Department of Emergency Medicine with complaints of excessive bleeding per vagina (4-5 pads soaked per day), which was associated with passage of clots and pain in abdomen for 4 days. One month prior, she has undergone dilatation and evacuation at 8 weeks of gestational age as ultrasonography was suggestive of missed abortion. Since then, she was observed to have bleeding per vagina, which could not be relieved on medication. At the time of presentation, bleeding became persistent and was found to be associated with extreme pain, dizziness, restlessness and weakness.

**Clinical findings:** on examination, the patient was pale and was hypotensive with tachycardia. On local examination, she presented with tenderness over the suprapubic region. On further examination per spectrum, cervical erosion was seen. On bimanual vaginal examination, the uterus was found to be bulky, anteverted, and ante flexed with bilateral fornices free.

**Timeline:** had a missed abortion about a month back for which she had undergone dilatation and evacuation. Since then, she presented with torrential bleeding per vaginum, not relieved by a medical method. She was then referred to the emergency department with complaints if breathlessness and extreme weakness.

**Figure 1:**
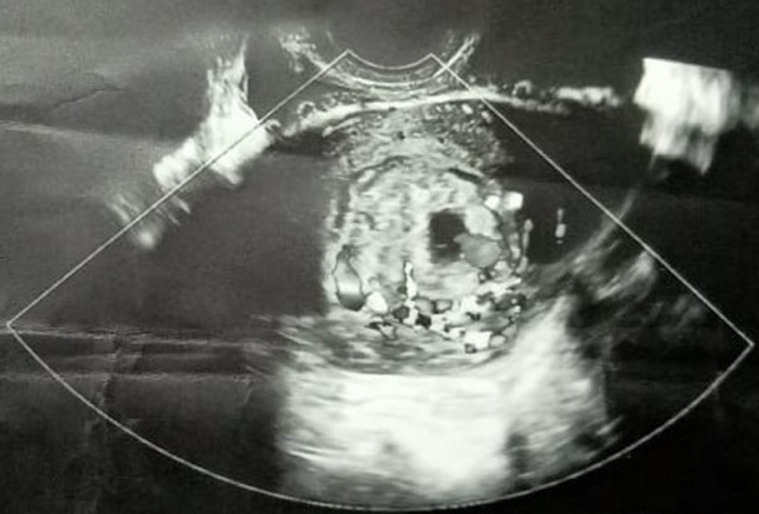
Doppler study showing vascular changes

**Figure 2:**
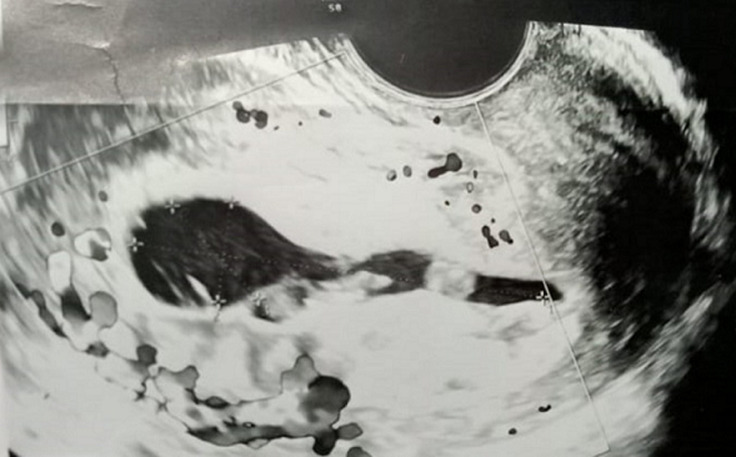
Doppler study demonstrating uterine arteriovenous malformation

**Figure 3:**
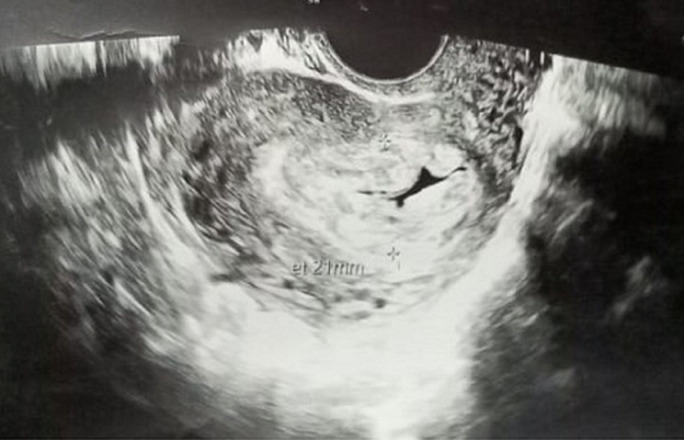
trans abdominal sonography showing endometrial thickness of 21 mm

**Figure 4:**
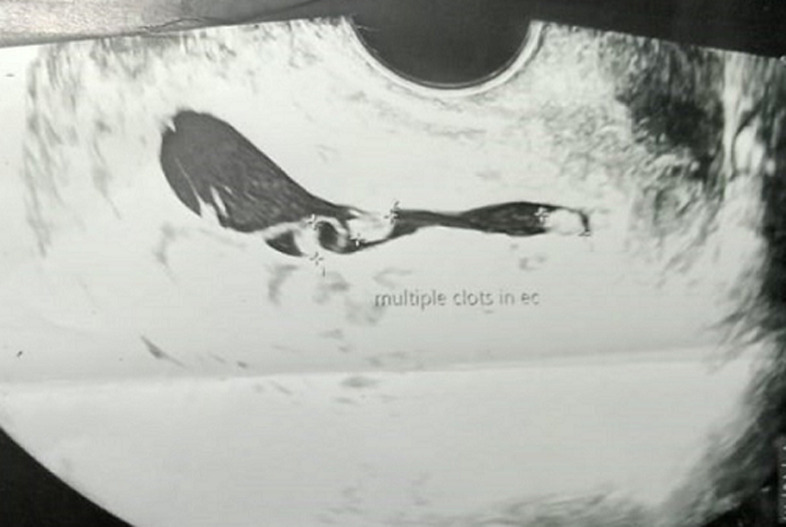
trans abdominal sonography demonstrating multiple clots in endometrial cavity

**Figure 5:**
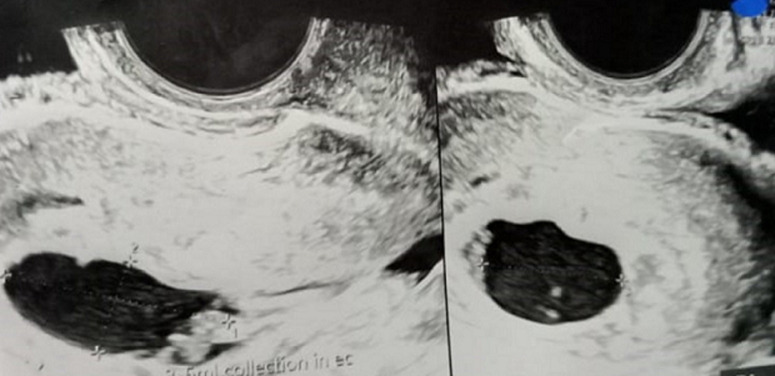
trans abdominal sonography showing 3-5 cc of collection in endometrial cavity

**Diagnostic assessment:** laboratory investigations revealed haemoglobin of 7.6gm/dl, platelet count of 60,000/cu mm, activated partial thromboplastin time (APTT) of 30.6 sec, Prothrombin Time of 13.8 sec and prothrombin time/international normalized ratio (PT/INR) found to be 1.1. Her beta-human chorionic gonadotropin (beta-HCG) was 180.34m IU/ml. On Doppler ultrasonography, the uterus was found to be of size 10.2 cms x 4.6 cms x 3.1 cms. Figure 1 depicts a Doppler study demonstrating vascular changes. Figure 2 shows a Doppler study demonstrating uterine arteriovenous malformation. There was evidence of an echogenic, heterogenous structure in the endometrial cavity. Figure 3 is of trans abdominal sonography, showing an endometrial thickness of 21 mm. Figure 4 shows trans abdominal sonography demonstrating multiple clots in the endometrial cavity. Vascularity on Doppler measuring 2.8mm x 2.5mm x 0.9mm, 3.6cc volume is noted. Figure 5 depicts trans abdominal sonography showing 3-5 cc of collection in the endometrial cavity.

**Figure 6:**
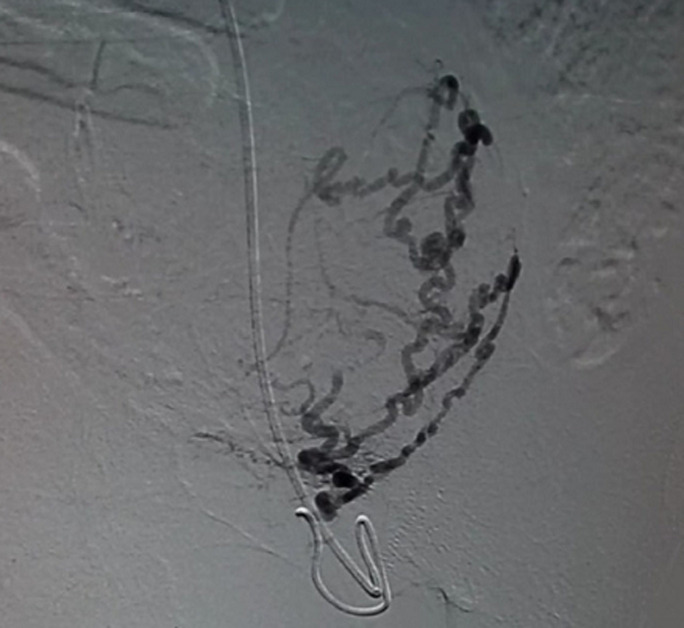
left uterine artery embolization

**Figure 7:**
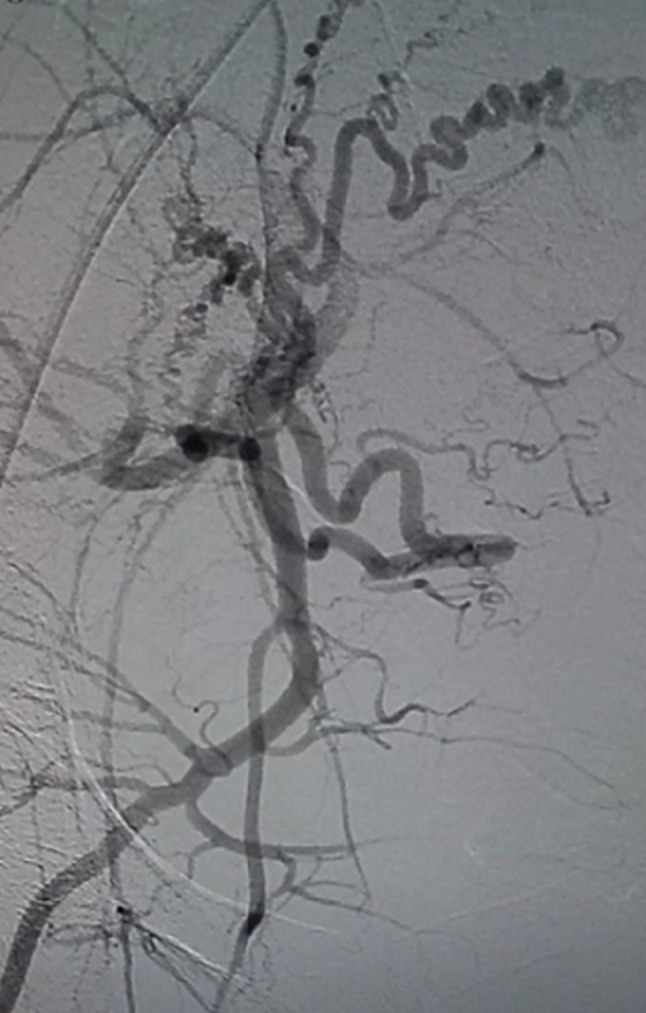
right uterine artery embolization

**Therapeutic intervention:** patient came with complaints of excessive bleeding per vagina to the Emergency Medicine Department. She was admitted to the high-dependency unit (HDU) where her vitals were taken and she was found to be hypotensive with a blood pressure of 92/60 mm Hg, and pulse rate 108 beats per minute. She was resuscitated with oxygen and intravenous fluids. She was initially managed medically for bleeding per vagina with tranexamic acid, but could not be subsided. Her haemoglobin was found to be 7.6 mg/dl and was given a transfusion with 2 units of human red blood cells (RBC). After being hemodynamically stable further management was planned. Patient being suspected of uterine AVM was then referred to the department of interventional radiology. There, the presence of AVM was confirmed by performing a uterine angiogram. Figure 6 demonstrates left uterine artery embolization Figure 7 shows right uterine artery embolization. Embolization of bilateral uterine arteries was carried out to near stasis. The post-embolization arteriogram demonstrated a slow flow of contrast in both uterine arteries, thereby confirming complete embolization of the AVM. There were no immediate complications encountered. The patient's vaginal bleeding was found to resolve. She was then discharged four days later.

**Follow-up and outcomes:** as per her planned follow-up care, after three months the patient visited the hospital. During the visit, she had no complaints of vaginal bleeding. The Doppler ultrasonography was conducted, and no vascularity was seen within the lesion, which demonstrated complete resolution of UAVM. Normal menstruation was resumed and the serum beta-human chorionic gonadotropin (beta-HCG) level was normalized. Thereafter, the patient was making thorough going to preparations for her next pregnancy.

**Patient perspective:** the patient was extremely satisfied with the timely therapeutic intervention made available. She is thankful for the amount of care and empathy shown by all the doctors, nurses, and staff.

**Informed consent:** an informed, verbal and written consent has been obtained from the patient.

## Discussion

Arteriovenous vascular malformation (AVMS) of the uterus, which could be either congenital or acquired, are uncommon but one of the most potentially life-threatening lesions [6]. Congenital AVMs may be the result of remnants of embryonic vascular connections. Acquired AVMs are usually more localized and are found in association with d and c, cesarean section, previous uterine surgery, malignancy, trophoblastic disease or diethylstilboestrol exposure [7]. The major presenting complaint is found to be hypermenorrhea often following invasive procedures such as therapeutic abortion or uterine curettage. In some of extreme cases, heavy uterine bleeding might even result in shock. Moreover, congestive cardiac failure-secondary to the vascular steal effect is another rare presentation. In mildly symptomatic patients, spontaneous remission of UAVM is predicted. Hence, it is essential to maintain regular radiologic investigations and clinical follow-up is mandatory for decisions regarding definitive management. As indicated in this case, if women of child bearing age group present with complaints of recurrent episodes of abnormal vaginal bleeding after dilation and curettage, UAVM should be explored as a possible cause. Menorrhagia is one of the most prevalent presenting symptoms. This is due to the fact that uterine bleeding occurs when the vasculature of AVMs are exposed due to endometrial sloughing during the menstrual cycle or iatrogenically following a dilation and curettage. In conditions of AVM, dilatation and curettage may result in a catastrophic outcome. Ultrasonography reveals numerous anechoic tortuous regions in the myometrium without mass impact, and hence it has become the investigation of choice in these patients [8].

To diagnose conditions like uterine AVM is challenging since it is a rare scenario and the routinely used diagnostic techniques, such as pelvic examination, followed by curettage, grey-scale ultrasonography or hysteroscopy generally fails to provide useful information. Traditionally, angiography has been used to confirm the diagnosis. Angiography is essential to demonstrate the vascular supply and is further used as a guide to performing action therapy [9]. Nonetheless, angiography is an invasive procedure, exposes the patient to radiation and is more time-consuming to perform. Recently, noninvasive techniques such as contrast enhanced CT (CECT), Magnetic resonance imaging (MRI) and color flow Doppler ultrasonography have been proposed to diagnose uterine AVMs. Treatment of uterine AVM is dependent on several parameters such as the amount of blood loss, the hemodynamic status, the patient´s age, and her wish for future fertility. Immediate management involves the arrest of blood loss followed by stabilizing the patient´s hemodynamic condition. Previously, hysterectomy was considered to be the treatment of choice in these conditions. However, as there are now new methods available to prevent hysterectomy, the patient's wish for future pregnancy plays an essential role in planning future management. Long-term medical management is considered appropriate in patients who are stable and who have the ability for regular follow-up. Furthermore, adopting methods such as intramuscular as well as oral contraception followed by the use of methylergonovine maleate have been found to be successful in causing regression of lesions according to ultrasonography findings [1]. Embolization technique for uterine AVM is generally used in fewer crisis circumstances as well as in emergency situations. Several embolic materials are being used, such as steel coils, polyvinyl alcohol, balloons-detachable, history (glue), and hemostatic gelatin. Rarely, few patients might require repeat embolization procedures [1]. In women of the reproductive age group, uterine AVM is generally diagnosed and has now made hysterectomy no longer necessary with the use of techniques like angiographic embolization. However, in an emergency setting and in post-menopausal age groups, a patient's hysterectomy continues to remain the treatment of choice [10].

## Conclusion

In all patients presenting with excessive bleeding following curettage or abortion, UAVM is considered to be one of the diagnostic possibilities. With the recent advancements in imaging modalities, the diagnosis of uterine AVM has come much simpler. Management by selective arterial embolization reduces the morbidity of surgery and hospital stay.
